# High-Frequency Ultrasound in the Assessment of Cellulite—Correlation between Ultrasound-Derived Measurements, Clinical Assessment, and Nürnberger–Müller Scale Scores

**DOI:** 10.3390/diagnostics14171878

**Published:** 2024-08-27

**Authors:** Robert Krzysztof Mlosek, Sylwia Patrycja Malinowska

**Affiliations:** 1 Diagnostic Ultrasound Laboratory, Medical University of Warsaw, 02-091 Warszawa, Poland; 2 Life-Beauty—Private Company, 05-825 Grodzisk Mazowiecki, Poland

**Keywords:** cellulite, high-frequency ultrasound, skin ultrasound, ultrasonography, aesthetic medicine

## Abstract

Background: Cellulite is a cosmetic defect of multifactorial etiology that affects over 90% of women worldwide. Cellulite-induced skin changes are undesirable and negatively affect self-esteem. Despite a plethora of cellulite-reducing treatments, we still lack objective tools to enable accurate diagnosis and treatment efficacy assessment. The aim of this study was to determine whether high-frequency ultrasound can be helpful in assessing cellulite and whether there is an association between ultrasound-derived measurements, parameters ascertained clinically, and cellulite assessment scale scores. Methods: The study group consisted of 114 women with cellulite in their posterior thighs, assessed using the Nürnberger–Müller scale. Two types of ultrasound devices were used in this study: a conventional scanner with a linear transducer and a skin-dedicated scanner equipped with a mechanical transducer. We used high-frequency ultrasonography to determine epidermal thickness, dermal thickness, the surface area of fat protrusions at the dermal subcutaneous junction, and the thickness and stiffness of the subcutaneous tissue (ultrasound elastography). Results: There was a correlation between cellulite severity and subcutaneous tissue thickness (r = 0.63), the surface area of fat protrusions at the dermal subcutaneous junction (r = 0.64), and the elastographic strain ratio (r = 0.51). An association was also demonstrated between thigh circumference and subcutaneous tissue thickness (r = 0.48). There was a significant difference in the assessed parameters between the subgroups identified by cellulite severity scores. Conclusions: Ultrasound-determined surface area of fat protrusions at the dermal subcutaneous junction as well as the thickness and stiffness of the subcutaneous tissue seem useful in cellulite assessment. Thus, ultrasonography has the potential to become a common tool in aesthetic medicine and cosmetology.

## 1. Introduction

Cellulite is the most common aesthetic problem, which affects 80–98% of all women, in particular those of Caucasian ethnicity [1,2]. Currently, cellulite is defined as non-inflammatory subcutaneous tissue degeneration of poorly understood etiology, which leads to the formation of edematous fibrosclerotic panniculopathy [1,2,3,4]. Clinical presentation of cellulite shows high variability, which poses a challenge upon diagnosis and grading. Cellulite develops in months-to-years-long stages, with blurred transitional boundaries between them. The initial stage is asymptomatic. It encompasses venous and lymphatic insufficiency alongside increased fat storage, causing adipocyte enlargement. At the second stage, microcirculatory abnormalities cause oedema and an uneven skin surface. As skin elasticity decreases, cellulite symptoms on its surface become more visible. At the next, more advanced stage, cellulite presents as visible pits, bumps, grooves, and painful lumps, which can coexist with telangiectasias, microvaricosity, stretch marks, skin discoloration (purple to yellow-gray) and different types of edema. These changes within the subcutaneous tissue present on the skin surface and give it a characteristic ‘mattress’-like or ‘orange peel’-like appearance [1,2,3,4].

Although not classified as a disease, cellulite reduction is considered desirable, as most women find it hard to accept the cellulite-induced changes in skin appearance and that it significantly impairs their quality of life [5]. Individuals with cellulite are very often ashamed of their physical appearance, which limits their options in daily life regarding dressing, physical activity, and even sexual life [6]. In response to the ubiquity of cellulite and its negative perception, cosmetology and aesthetic medicine have developed an ever-increasing range of cellulite-reduction methods and treatments. According to the Future Market Insights report presenting the global cellulite management market analysis 2013–2021 and forecast 2022–2028, the value of the market is growing steadily [7]. By 2028, it is projected to increase by a further 8% compared to 2022, reaching a revenue of USD 5.2 billion. Cellulite reduction methods and treatments range from systemic oral agents and topical therapies based on the delivery of selected active ingredients to procedures using modern devices and surgical approaches [2,3,4,8,9]. Thus, cellulite is the shared focus of cosmetologists, physiotherapists, and physicians. Unfortunately, many of the available cosmetic products, therapies, and cellulite reduction methods have no proven efficacy, and any evidence to support them is based on subjective assessment methods, as there is no legal requirement for the manufacturers of skin care products or devices to conduct in vivo studies. To date, there has been a scarcity of research to assess the efficacy of selected cellulite reduction methods. Furthermore, comparing the results of existing studies is often impossible due to differences in treatment protocols and study designs [1,10]. The challenge lies not only in assessing treatment efficacy but also in diagnosing cellulite and assessing its severity [1,3,4,5,10,11]. Cellulite diagnosis is made based on case history, clinical examination, and assessment using specialist equipment, such as thermography, different diagnostic imaging techniques, and bioelectrical impedance analysis [3,10,11]. In contact thermography, aluminum foil is applied onto the surface of the skin. Heat transfer occurs between the skin surface and the foil, whereby a thermographic pattern is created depending on skin temperature in different areas, which reflects microcirculation. Cellulite-affected skin tends to have a lower temperature, so it will be represented by darker colors on the thermographic map. While the method is inexpensive and simple, the results may be affected by such external factors as sun exposure, the menstrual cycle, or the patient’s psychophysical arousal. Non-contact thermography, based on capturing thermal energy emitted by the human body, enables more objective measurements. However, it requires more effort to prepare the patient for imaging and to ensure appropriate imaging conditions, which can be challenging [12]. Bioelectrical impedance analysis, which measures the resistance and reactance of individual tissues, is also used for cellulite assessment and cellulite-reduction treatment evaluation. As the impedance varies between the tissue types, it is possible to estimate body composition by determining the percentage content of adipose tissue, muscles, and water. While the technique itself is simple, it carries the same risk as thermography; that is, any comparisons and conclusions may be unreliable, as it is very difficult to ensure consistent assessment conditions. Diagnostic imaging techniques, such as magnetic resonance imaging (MRI), computed tomography (CT), and ultrasonography (US), enable the most reliable measurements. The MRI is considered a reliable adipose tissue assessment, which enables accurate imaging of both dermis and subcutaneous tissue, including connective tissue septa. The MRI offers high sensitivity and specificity, so it can objectively assess cellulite before and after treatment. The CT, on the other hand, enables adipose thickness measurement without assessing the dermis. The limitations of both methods include their low availability, high cost, and X-ray exposure from CT scans. Considering the above, the US appears to be the only imaging modality with the potential for widespread use in cellulite assessment [11,13,14,15,16].

The ultrasound cellulite assessment can be carried out using both classical scanners equipped with high-frequency linear transducers as well as dedicated skin scanners, typically equipped with 20–100 MHz mechanical single-element transducers. In this study, we used both types of ultrasound devices to obtain ultrasound images of cellulite-affected skin. It provides information on the thickness of individual skin layers, the epidermis, the dermis, and the subcutaneous tissue. It also enables assessing the surface area or the length of fat protrusions (called papillae adiposae) at the dermal subcutaneous junction [15]. Furthermore, subcutaneous tissue stiffness can be assessed using ultrasound elastography. Our research as well as studies published by other authors confirm its usefulness in cellulite assessment [13,14,15,16]. Thus, considering that ultrasonography is an objective, reproducible, reliable, safe, non-invasive, and relatively low-cost method, it has the potential to become popular in cellulite assessment and monitoring the efficacy of cellulite-reduction treatments. Notably, ultrasonography is the only non-invasive modality that enables “peering into” the skin and that is not affected by assessment conditions or patient preparation, which renders it superior to other diagnostic methods, such as thermography or bioelectrical impedance analysis. Furthermore, the advantage of ultrasonography over other diagnostic techniques stems from the fact that ultrasound technologies have been growing exponentially in recent years. Mobile ultrasound scanners have become available, which offer increasingly higher-quality imaging and have the potential to become commonly used in practices treating patients with cellulite. This supports the need for quality research to become a basis for the standardized ultrasound-based cellulite assessment and treatment monitoring protocols, as well as uniform anti-cellulite treatment standards, as there is currently no “gold standard” in any of the three above areas.

The aim of our study was to determine whether high-frequency ultrasound is useful for cellulite assessment. We also aimed to determine whether there were correlations between ultrasound-ascertained measurements, clinical assessment findings, and cellulite severity scores assessed using a dedicated rating scale.

## 2. Materials and Methods

The series of ultrasound assessments of individuals with cellulite were carried out in 2016–2022 as a part of the research approved by the Ethics Committee of the Medical University of Warsaw (approval number: KB/67/2014). Study participants were recruited from volunteers who met the basic inclusion criterion of cellulite presence in their posterior thighs. We reviewed those ultrasound scans, retrospectively, in January–March 2024 and selected 114 females to be enrolled in the current study group. The study protocol was compliant with the Declaration of Helsinki.

### 2.1. Study Group and Clinical Assessment

The study group consisted of 114 females aged 18–67 years (mean age = 42.08 years, standard deviation (SD) = 10.99). All participants had cellulite on the posterior aspect of their thighs. Each participant was administered a structured interview to ascertain their general health, eating habits, lifestyle, and physical activity, as well as the duration of cellulite and previously used anti-cellulite treatments. The exclusion criteria included pregnancy, breastfeeding, diabetes, malignancies, severe varicose veins, and anti-cellulite treatment used within 6 weeks prior to enrollment. Cellulite was confirmed by the positive pinch test. The pinch test involved pinching skin on the posterior surface of the thigh between the thumb and index finger. An orange peel-like or mattress-like appearance of the skin upon pinching confirmed the presence of cellulite. Biometric measurements (body weight, body height, and thigh circumference) were also carried out. Body weight and height were assessed using Sid-65 medical scales (Sideon, China), and thigh circumference was measured using an anthropometric tape. The body mass index (BMI) was calculated for each participant, and the pinch test findings were assessed using the Nürnberger–Müller scale to determine cellulite severity [17]. The Nürnberger–Müller scale categorizes cellulite into 4 grades based on skin appearance:

- grade 0—no cellulite, skin is smooth on pinching,
- grade I—skin is smooth at rest, but shows a mattress-like appearance upon pinching,
- grade II—skin is smooth at rest, but shows a mattress-like appearance upon standing,
- grade III—the skin has a mattress-like appearance in both the lying and standing positions.

It demonstrated that 24 women had grade I cellulite (mean age = 43.78 years, SD = 13.32), 59 women had grade II cellulite (mean age = 41.34 years, SD = 10.90), whereas 31 women had grade III cellulite (mean age = 42.16 years, SD = 9.47).

### 2.2. Ultrasound Assessment

Each ultrasound assessment was carried out using the Philips Epiq 5 scanner (Philips, USA) using the 18 MHz linear transducer (model L18-5), with the option of ultrasound elastography, as well as using the DermaMed skin-dedicated ultrasound device (Draminski, Dramiński, Olsztyn, Poland) using the 48 MHz mechanical single-element transducer. All ultrasound scans were taken at the same anatomical location on the posterior surface of the thigh. The exact location was marked on the skin, and the photo was taken to reproduce it for subsequent assessments. Normally it was a midpoint within the posterior thigh, unless an individual had any birth marks or scars elsewhere on the posterior surface of the thigh, in which case the assessment point was identified in their proximity to enable locating it more easily for subsequent assessments. All ultrasound assessments were carried out by the authors.

Using the conventional ultrasound scanner with the tissue strain elastography (static elastography/compression elastography) option, we measured the thickness of the subcutaneous tissue, and its stiffness compared to the underlying muscle (elastographic strain ratio). Each subcutaneous tissue measurement was taken twice, and the mean of those two values was used for analysis. In order to determine subcutaneous tissue stiffness, the ultrasound images were assessed, and two regions of interest (ROI) were identified to obtain quantitative data. The first ROI was within the subcutaneous tissue, and the second one was within the underlying muscle. Thus, within-subject comparisons between the two ROIs were made. Based on our previous research, we hypothesized that cellulite severity would not affect muscle stiffness, but it would affect subcutaneous tissue stiffness [18]. Thus, we hypothesized that the subcutaneous tissue of individuals with less severe cellulite would be firmer, harder, and stiffer than in those with more severe cellulite. Using the device-embedded software, we determined the elastographic strain ratio by dividing the strain value of the subcutaneous tissue by the mean strain value of the underlying muscle. In order to minimize possible measurement error, all ultrasound elastography assessments were carried out by the same clinician; each parameter was ascertained twice, in two consecutive measurements, and the mean of those two measurements was used for analyses.

Using the ultrasound scanner with a mechanical transducer, we determined epidermis and dermis thickness, as well as the surface area of fat protrusions at the dermal subcutaneous junction. Each measurement was taken twice, and the mean of those two measurements was used for analysis.

The ultrasound scanner settings were identical for all ultrasound assessments carried out as part of the study. The Philips Epiq 5 scanner had a gain of 60%, a dynamic range 68, and a mechanical index of 1.0. The Derma Med scanner had a near field gain of 25, an intermediate field gain of 25, and a far field gain of 25.

### 2.3. Statistical Analyses

The data was analyzed using the Statistica 13.3 (TIBCO Software Inc., Palo Alto, CA, USA) software bundle. Descriptive statistics were calculated for all variables. Distribution normality was ascertained using the Shapiro–Wilk test. A univariate ANOVA was carried out to analyze normally distributed variables, and the non-parametric Kruskal–Wallis test by ranks was used to analyze non-normally distributed variables. Correlations between the assessed variables were determined using the Pearson (for normally distributed variables) and Spearmann (for non-normally distributed variables) correlation coefficients. Guildford’s Rule of Thumb was adopted for interpreting the strength of correlations. The α = 0.05 was considered statistically significant.

## 3. Results

The collected data made it possible to determine cellulite severity and analyze correlations between the ultrasound-assessed and clinically assessed variables. We analyzed our results for the entire study group (*n* = 114) and for subgroups identified based on cellulite severity assessed using the Nürnberger–Müller scale. Participants with grade I, grade II, and grade III cellulite were allocated to group 1 (*n* = 24), group 2 (*n* = 59), and group 3 (*n* = 31), respectively.

### 3.1. Ultrasound Cellulite Assessment

#### 3.1.1. Epidermis Thickness

**Figure 1:**
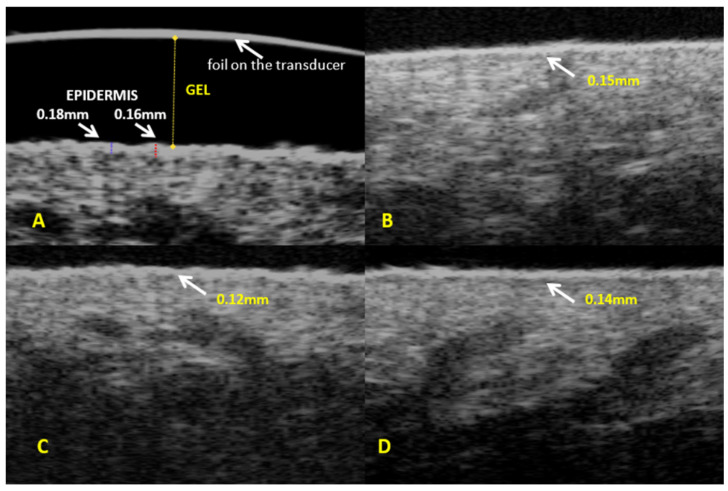
Ultrasound assessment of epidermis—(**A**). Epidermis thickness: (**B**)—group 1, (**C**)—group 2, (**D**)—group 3.

The mean epidermis thickness measured using high-frequency ultrasound in the entire study sample (*n* = 114) was 0.13 mm, SD = 0.03 mm (Table 1). Subgroup analysis demonstrated that the mean epidermis thickness was similar in three subgroups identified based on cellulite severity (0.12 mm, 0.13 mm, and 0.13 mm in groups 1, 2, and 3, respectively) with no significant between-group differences (Table 2, Figure 1 and Figure S1).

#### 3.1.2. Dermis Thickness

**Figure 2:**
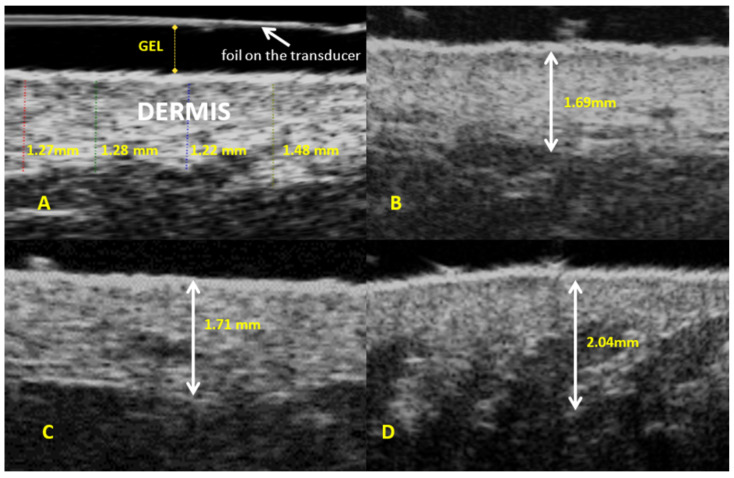
Ultrasound assessment of dermis (**A**). Dermis thickness: (**B**)—group 1; (**C**)—group 2; and (**D**)—group 3.

The mean dermis thickness measured using the scanner equipped with a 48 MHz mechanical transducer in the entire study sample (*n* = 114) was 1.54 mm, SD = 0.25 mm (Table 1). Subgroup analysis demonstrated the lowest mean dermis thickness in group 1 (1.45 mm) and the highest mean dermis thickness in group 2 (1.57 mm) (Figure 2 and Figure S2). The between-group differences were not significant, though, as demonstrated by ANOVA (Table 2).

#### 3.1.3. Surface Area of Fat Protrusions at the Dermal Subcutaneous Junction

**Figure 3:**
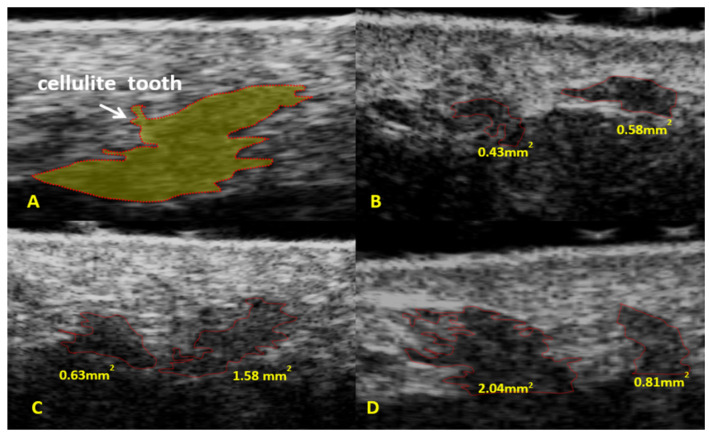
Ultrasound assessment of fat protrusions at the dermal subcutaneous junction (**A**). Surface area of fat protrusions at the dermal subcutaneous junction: (**B**)—group 1; (**C**)—group 2; and (**D**)—group 3.

The mean surface area of fat protrusions at the dermal subcutaneous junction in the entire study sample (*n* = 114) was 0.93 mm2 (Table 1). Subsequently, the between-group differences for groups 1–3 were analyzed (Figure 3 and Figure S3, Table 2). The smallest mean surface area of fat protrusions at the dermal subcutaneous junction was demonstrated in group 1, consisting of females with grade I cellulite assessed using Nürnberger–Müller scale (0.55 mm^2^). The largest mean surface area of fat protrusions at the dermal subcutaneous junction was demonstrated in group 3, consisting of females with grade III cellulite assessed using Nürnberger–Müller scale (1.44 mm^2^). The mean surface area of the dermal subcutaneous junction in group 2 was 0.82 mm^2^. The difference between groups 1 and 3 was significant (H = 48.27, *p* ≤ 0.001) (Table 2).

#### 3.1.4. Subcutaneous Tissue Thickness

**Figure 4:**
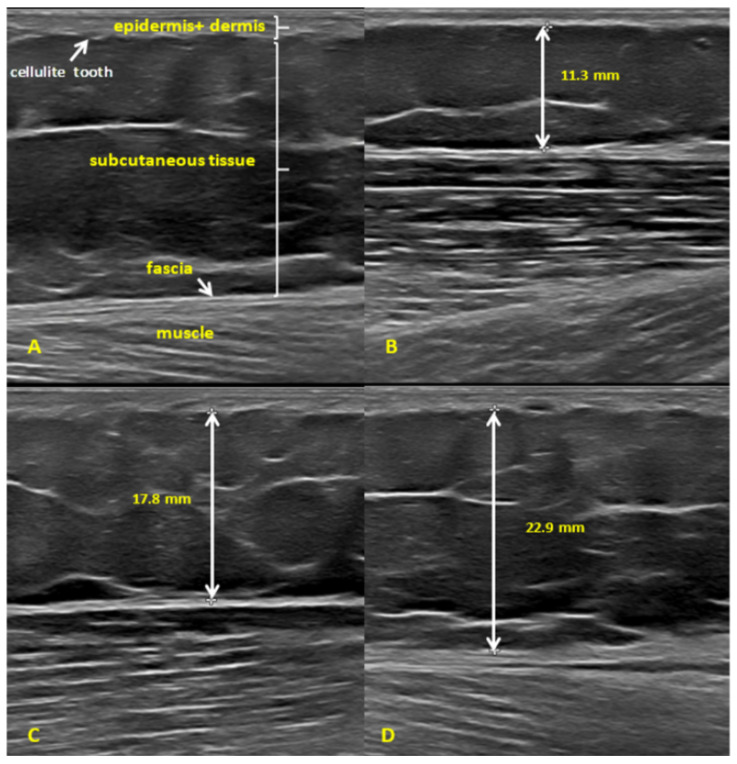
Ultrasound assessment of subcutaneous tissue (**A**). Subcutaneous tissue thickness: (**B**)—group 1; (**C**)—group 2; and (**D**)—group 3.

The mean subcutaneous tissue thickness measured at the posterior thigh using the conventional scanner in the entire study sample (*n* = 114) was 18.05 mm (Table 1). Subsequently, the between-group differences for groups 1–3 were analyzed (Figure 4 and Figure S4, Table 2). The mean subcutaneous tissue thickness was 12.48 mm, 15.85 mm, and 26.56 mm in groups 1, 2, and 3, respectively (Table 2). The between-group differences in subcutaneous tissue thickness expressed as percentage were 27% (group 1 vs. group 2), 67.5% (group 2 vs. group 3), and 112.82% (group 1 vs. group 3). All between-group differences were significant, as demonstrated by the non-parametric Kruskal–Wallis test by ranks (H = 47.51, *p* ≤ 0.001).

#### 3.1.5. Ultrasound Elastography of Subcutaneous Tissue

**Figure 5:**
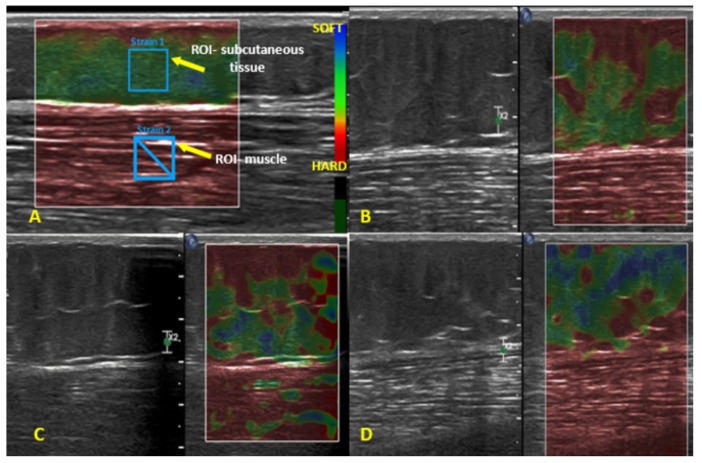
Ultrasound elastography of subcutaneous tissue (**A**). Ultrasound elastography of subcutaneous tissue: (**B**)—group 1; (**C**)—group 2; and (**D**)—group 3.

The mean elastographic strain ratio in the entire study sample (*n* = 114) was 2.06 (Table 1). Subsequently, the between-group differences for groups 1–3 were analyzed (Figure 5 and Figure S5, Table 2). The mean elastographic strain ratio was 1.51, 1.88, and 2.75 in groups 1, 2, and 3, respectively. Thus, the between-group differences expressed as percentages were 24.5% (group 2 vs. group 1), 46.28% (group 3 vs. group 2), and 82.12% (group 3 vs. group 1). Participants from group 1 had the firmest (stiffest, i.e., least susceptible to strain) subcutaneous tissue. All between-group differences were significant (H = 31.43, *p* ≤ 0.001) (Table 2).

### 3.2. Clinically-Ascertained Parameters

The clinically ascertained parameters included thigh circumference, body weight, BMI, and cellulite severity assessment using the Nürnberger–Müller scale. The mean thigh circumference in the entire study sample (*n* = 114) was 55.04 cm (Table 1). For subgroup analysis, the mean values in groups 2 and 3 were comparable, while group 1 had the mean thigh circumference of 50.88 cm, which was significantly lower than in both remaining groups (F = 9.04, *p* ≤ 0.001) (Table 2).

There were no significant between-group differences in body weight or BMI. The mean body weight and the mean BMI in the entire study sample (*n* = 114) were 67.01 kg and 23.82, respectively. It shows that our study participants had a normal body weight. The mean cellulite severity assessed using the Nürnberger–Müller scale in the entire study sample (*n* = 114) was 2.06 (Table 1). All enrolled participants had cellulite, so the severity ranged between 1 and 3.

### 3.3. Correlation Analysis

The collected data enabled correlational analysis. Thus, correlations were assessed between variables ascertained using an ultrasound scanner equipped with a mechanical transducer (epidermis thickness, dermis thickness, and surface area of fat protrusions at the dermal subcutaneous junction), variables ascertained using a conventional ultrasound scanner (subcutaneous tissue thickness and elastographic strain ratio), and variables ascertained in clinical assessment (thigh circumference, body weight, BMI, and cellulite severity assessed using the Nürnberger–Müller scale) (Table 3). Guildford’s Rule of Thumb was adopted for interpreting the strength of correlations.

The strongest correlation was identified between cellulite severity and ultrasound-derived parameters. A moderately significant (*p* ≤ 0.001) correlation was identified between cellulite severity assessed using the Nürnberger–Müller scale and the surface area of fat protrusions at the dermal subcutaneous junction (r = 0.64), subcutaneous tissue thickness (r = 0.63), and elastographic strain ratio (r = 0.51), respectively. All of the above were positive correlations, which means that the higher cellulite severity grade is associated with a larger surface area of fat protrusions at the dermal subcutaneous junction, increased subcutaneous tissue thickness, and an increased elastographic strain ratio.

As for dermis thickness, it showed a moderately significant correlation with subcutaneous tissue thickness (r = 0.48, *p* ≤ 0.001) and a low significant correlation with thigh circumference (r = 0.39, *p* ≤ 0.001). Additionally, a low, significant correlation was identified between thigh circumference and elastographic strain ratio (r = 0.20, *p* ≤ 0.001). Nevertheless, we excluded all correlations with a strength below r = 0.4 from further analyses. No other significant correlations between the studied variables were identified.

## 4. Discussion

Given the prevalence of cellulite and the limitations it may imply, it has become important to develop global standards for the assessment of cellulite and monitoring its treatment effects [1,2,3,4,5,6,8,9,10]. Therefore, the aim of the current study was to determine whether high-frequency ultrasound can be helpful in assessing cellulite, to identify useful and reliable assessment parameters, and to assess the associations between ultrasound-derived measurements, parameters ascertained clinically, and cellulite assessment scale scores.

In line with ultrasound physics, the higher the ultrasound frequency, the less it penetrates into the tissue. Thus, 10 MHz, 20 MHz, and 50 MHz transducers offer tissue penetration of about 35 mm, 10 mm, and 3–4 mm, respectively [19]. Consequently, accurate cellulite imaging will require different transducer and ultrasound scanner types, which enable visualizing both superficial (e.g., epidermis) and slightly deeper (e.g., subcutaneous tissue) structures. The need for different types of diagnostic equipment has been a significant barrier to the uptake of ultrasonography in dermatology, including for cellulite assessment. However, with the rapid development of ultrasound imaging platforms, including mobile devices, which work with different types of transducers, we can expect this barrier to be removed in the near future as ultrasound scanners become even more readily available and commonly used.

The unique aspect of the current study involves the use of two different ultrasound scanner types. This has not been attempted elsewhere except for our own previously published research [15,18]. The conventional scanner, equipped with a linear transducer, enabled subcutaneous tissue measurements and the calculation of the elastographic strain ratio. The scanner with a 48 MHz mechanical transducer, which offers relatively shallow tissue penetration, enabled high-resolution images of the epidermis and dermis, as well as assessing the surface area of fat protrusions at the dermal subcutaneous junction. And it is the assessment of that junction, and more specifically of fat protrusions, which develop in cellulite-affected skin, that we believe to be most useful in such individuals.

The fat protrusions seen in an ultrasound at the dermal subcutaneous junction, which create a tethering effect typical of cellulite, have been previously described by other authors [15,18,20,21,22]. In cellulite-free skin, the interface between the dermis and subcutaneous tissue has a straight, linear appearance. Some researchers assessed its thickness, demonstrating that it becomes thicker with increasing cellulite severity [11,20,22,23]. However, as demonstrated by Soares et al. [14], the correlation between instrumental and clinical parameters in patients with cellulite was poor. We felt that dermal subcutaneous junction thickness measurement was a technically difficult one. Therefore, in search of a suitable alternative, we previously attempted to measure the length of fat protrusions in this area [15,18]. This, nevertheless, proved at least equally difficult, additionally posing a high risk of measurement error. In the current study, we slightly modified our approach and used the dedicated scanner software bundle to determine the surface area of fat protrusions at the dermal subcutaneous junction. Compared to the junction thickness and length of fat protrusions, this seems to be the easiest and fastest measurement possible. Furthermore, fat protrusions differ in echogenicity from the dermis, which makes them easier to visualize. Our findings demonstrated a moderate correlation between the surface area of fat protrusions at the dermal subcutaneous junction and clinical assessment of cellulite severity.

Subcutaneous tissue thickness appears as another useful parameter in cellulite assessment. We found a strong correlation between subcutaneous tissue thickness and the clinical assessment of cellulite severity. The thickness increases with an increase in cellulite grading as per the Nürnberger–Müller scale. We also found another positive correlation between subcutaneous tissue thickness and thigh circumference. To our knowledge, there has been no other published study that we could directly compare our results to. Indirectly, however, we can discuss our findings in the context of studies investigating the correlation between cellulite severity and the thickness of the fat fold in the cellulite-affected area measured using the skinfold caliper [20,24]. Kołodziejczak et al. [24] demonstrated a strong positive correlation between the fat fold thickness and Nürnberger–Müller scale score.

Ultrasound elastography also seems useful in cellulite assessment. Our results demonstrated that subcutaneous tissue stiffness was associated with cellulite severity. The lower stiffness predicted a higher grade of cellulite. Again, we could not identify another published study to directly compare our results to. However, ultrasound elastography has been previously shown to be effective in dermatology and aesthetic medicine in the assessment of both healthy and abnormal skin [25,26]. The use of the elastographic strain ratio needs further research, not only due to the scarcity of published data. In our study, we opted for static elastography, the accuracy of which is strongly linked to the learning curve and depends on the measuring clinician, so as such, it may lack objectivity. Newer reports suggest that shear wave elastography offers more objective measurements [25,26].

Epidermis and dermis thickness proved to be of no use in ultrasound cellulite assessment. Similarly, there was no correlation between any of the assessed parameters and body weight or BMI. Similarly, Soares et al. [14] reported a lack of correlation between body weight, BMI, and dermis thickness.

The results obtained in this study have allowed us to identify ultrasonographic parameters that are useful in assessing cellulite. Each of these parameters can be used not only to evaluate the presence or absence of cellulite but also to assess the effectiveness of anti-cellulite therapies [11,14,15,18,21]. In this case, ultrasonographic measurements are taken before and after the completion of therapy and then compared with each other. Ultrasound allows for the assessment of changes in the skin affected by cellulite following the application of any type of anti-cellulite therapy. The most commonly used parameter in this regard is the measurement of the surface area of fat protrusions at the dermal subcutaneous junction. A therapy is considered effective if it leads to a reduction in the surface area of fat protrusions at the dermal subcutaneous junction and an improvement in the appearance of the skin affected by cellulite [14,15,18,21]. Performing an ultrasonographic measurement (after appropriate training) is relatively easy, quick, and allows for obtaining reliable measurements. Therefore, it can have significant applications in the daily practice of cosmetologists and doctors involved in anti-cellulite therapies. However, it is essential to develop a standardized procedure for conducting such an examination to ensure that the results are reliable and comparable.

To sum up, our results support high-frequency ultrasound as an effective cellulite imaging method and enable identifying useful ultrasound-ascertained parameters. Those conclusions are supported by other authors [14,15,16,17,18,19,20,21], who similarly suggested that ultrasound imaging can be successfully used in cellulite assessment. Furthermore, high-frequency ultrasound can prove useful in real-life monitoring of cellulite-reducing treatments, such as subcision, which improves the efficacy and safety of such treatments [27].

Nevertheless, our study had certain limitations. The first of them is associated with the Nürnberger–Müller scale, which we decided to use in this study. While it is one of the most popular and commonly used cellulite severity scales, it does not cover all cellulite symptoms. Some authors suggest that this may make such a severity assessment less reliable [11]. This is also why the Nürnberger–Müller scale has been gradually replaced by the photonumeric cellulite severity scale in recent years [28]. The latter scale was also used by Whipple et al. [27], who demonstrated a positive correlation between cellulite severity, “depth” of cellulite dimples, and body weight. Although the photonumeric cellulite severity scale offers improved reliability and accuracy, it does so at the expense of increased complexity, which makes it difficult to use in day-to-day clinical practice [11].

Further research is, therefore, warranted. Another limitation is associated with our study sample consisting of only females with cellulite. As a result, we have no data on ultrasound image and correlation of investigated parameters in cellulite-free skin. Further research may enable developing an ultrasound-based severity assessment protocol and scale. This would, in turn, inform the global cellulite diagnostic assessments standards, applicable to both research projects and clinical practice.

## 5. Conclusions

High-frequency ultrasound is an effective tool in cellulite assessment. Both conventional devices equipped with linear transducers as well as those with skin-dedicated mechanical transducers can be used. Cellulite assessment can incorporate several useful ultrasound-derived parameters, such as the surface area of subcutaneous fat protrusions, subcutaneous tissue thickness, and subcutaneous ultrasound elastography. The increasing severity of cellulite is associated with an increased surface area of subcutaneous fat protrusions, increased subcutaneous tissue thickness, and lower stiffness.

Since ultrasonography is a non-invasive, safe, and reliable diagnostic imaging modality, it has the potential to become a popular tool for cellulite assessment in aesthetic medicine and cosmetology. This supports the need for further research in order to develop a uniform ultrasound-based cellulite severity scale and a dedicated assessment protocol.

## Figures and Tables

**Table 1:** Results of skin measurements affected by cellulite in the entire study sample.

PARAMETRS		$\overset{-}{x}$	Min	Max	SD
High-frequency ultrasound	Epidermis thickness [mm]	0.13	0.08	0.20	0.03
	Dermis thickness [mm]	1.54	0.83	2.18	0.25
	Surface area of fat protrusions [mm^2^]	0.93	0.20	2.58	0.50
Classical ultrasound	Subcutaneous tissue thickness [mm]	18.05	5.25	36.80	7.53
	Ultrasound elastography	2.04	0.46	4.88	0.88
Clinically ascertained parametrs	Thigh circumference [cm]	55.04	37.00	69.00	5.80
	Body weight [kg]	67.01	47.00	98.00	9.59
	BMI	23.82	17.47	38.76	3.51
	Cellulite severity (Nürnberger–Müller scores)	2.06	1.00	3.00	0.69

$\overset{-}{x}$—arithmetic mean; Min—minimum value; Max—maximum value; SD—standard deviation.

**Table 2:** Results of skin measurements affected by cellulite in groups 1–3.

PARAMETRS		GRADE I CELLULITE *n* = 24				GRADE II CELLULITE *n* = 59				GRADE III CELLULITE *n* = 31				ANOVA (F)/ANOVA Kruskala-Wallisa (H)	
		$\overset{-}{x}$	Min	Max	SD	$\overset{-}{x}$	Min	Max	SD	$\overset{-}{x}$	Min	Max	SD	F/H	*p*
High-frequency ultrasound	Epidermis thickness [mm]	0.12	0.08	0.15	0.02	0.13	0.09	0.20	0.03	0.13	0.08	0.19	0.03	H = 1.57	0.455
	Dermis thickness [mm]	1.45	0.83	2.02	0.32	1.57	1.07	2.18	0.24	1.55	1.18	2.04	0.19	F = 1.87	0.157
	Surface area of fat protrusions [mm^2^]	0.55	0.20	0.91	0.19	0.82	0.20	1.63	0.34	1.44	0.65	2.58	0.53	H = 48.27	*p* ≤ 0.001
Classical ultrasound	Subcutaneous tissue thickness [mm]	12.48	5.25	18.50	3.74	15.85	7.02	31.70	4.95	26.56	12.80	36.80	6.80	H = 47.51	*p* ≤ 0.001
	Ultrasound elastography	1.51	0.46	3.04	0.70	1.88	0.75	4.88	0.74	2.75	0.97	4.33	0.85	H = 31.43	*p* ≤ 0.001
Clinically ascertained parameters	Thigh circumference [cm]	50.88	37.00	60.00	5.67	55.97	45.00	69.00	5.41	56.48	47.00	67.00	5.27	F = 9.04	*p* ≤ 0.001
	Body weight [kg]	62.77	47.00	80.00	8.37	67.44	51.00	98.00	9.55	69.49	55.00	94.00	9.75	H = 5.73	0.057
	BMI	22.44	17.47	28.69	2.95	24.11	17.65	38.76	3.71	24.32	19.16	33.71	3.33	H = 5.81	0.055

*n*—simple size; $\overset{-}{x}$—arithmetic mean; Min—minimum value; Max–maximum value; SD—standard deviation; F—ANOVA value; H—Kruskal–Wallis ANOVA value; *p*—statistical significance level. Statistically significant results are marked in red.

**Table 3:** Correlations (Spearman’s R coefficient) between ultrasound examinations and clinically ascertained parameters.

PARAMETERS	Sample Size	Thigh Circumference [cm]	Body Weight [kg]	BMI	Cellulite Severity (Nürnberger–Müller Scores)
Epidermis thickness [mm]	114	0.01	−0.01	0.03	0.07
		*p* = 0.979	*p* = 0.891	*p* = 0.777	*p* = 0.462
Dermis thickness [mm]	114	0.39	−0.01	0.05	0.10
		*p* ≤ 0.001	*p* = 0.901	*p* = 0.613	*p* = 0.289
Surface area of fat protrusions [mm^2^]	114	0.16	0.09	0.08	0.64
		*p* = 0.086	*p* = 0.323	*p* = 0.381	*p* ≤ 0.001
Subcutaneous tissue thickness [mm]	114	0.48	0.11	0.11	0.63
		*p* ≤ 0.001	*p* = 0.229	*p* = 0.243	*p* ≤ 0.001
Ultrasound elastography	114	0.20	−0.01	0.01	0.51
		*p* ≤ 0.001	*p* = 0.943	*p* = 0.986	*p* ≤ 0.001

*p*—statistical significance level. Statistically significant results are marked in red.

## Data Availability

The data presented in this study are available upon request from the corresponding author. The data are not publicly available due to privacy reasons.

## Supplementary Materials

The following supporting information can be downloaded at: https://www.mdpi.com/article/10.3390/diagnostics14171878/s1. Figure S1. Epidermis thickness in groups 1–3. Figure S2. Dermis thickness in groups 1–3. Figure S3. Surface area of fat protrusions at the dermal subcutaneous junction in groups 1–3. Figure S4. Subcutaneous tissue thickness in groups 1–3. Figure S5. Ultrasound elastography of subcutaneous tissue in groups 1–3.
