# Supplementary figures and images for: High-Frequency Ultrasound in the Assessment of Cellulite—Correlation between Ultrasound-Derived Measurements, Clinical Assessment, and Nürnberger–Müller Scale Scores
Source: Diagnostics (Basel). 2024 Aug 27;14(17):1878. doi: 10.3390/diagnostics14171878 (PMC11393914; doi:10.3390/diagnostics14171878)

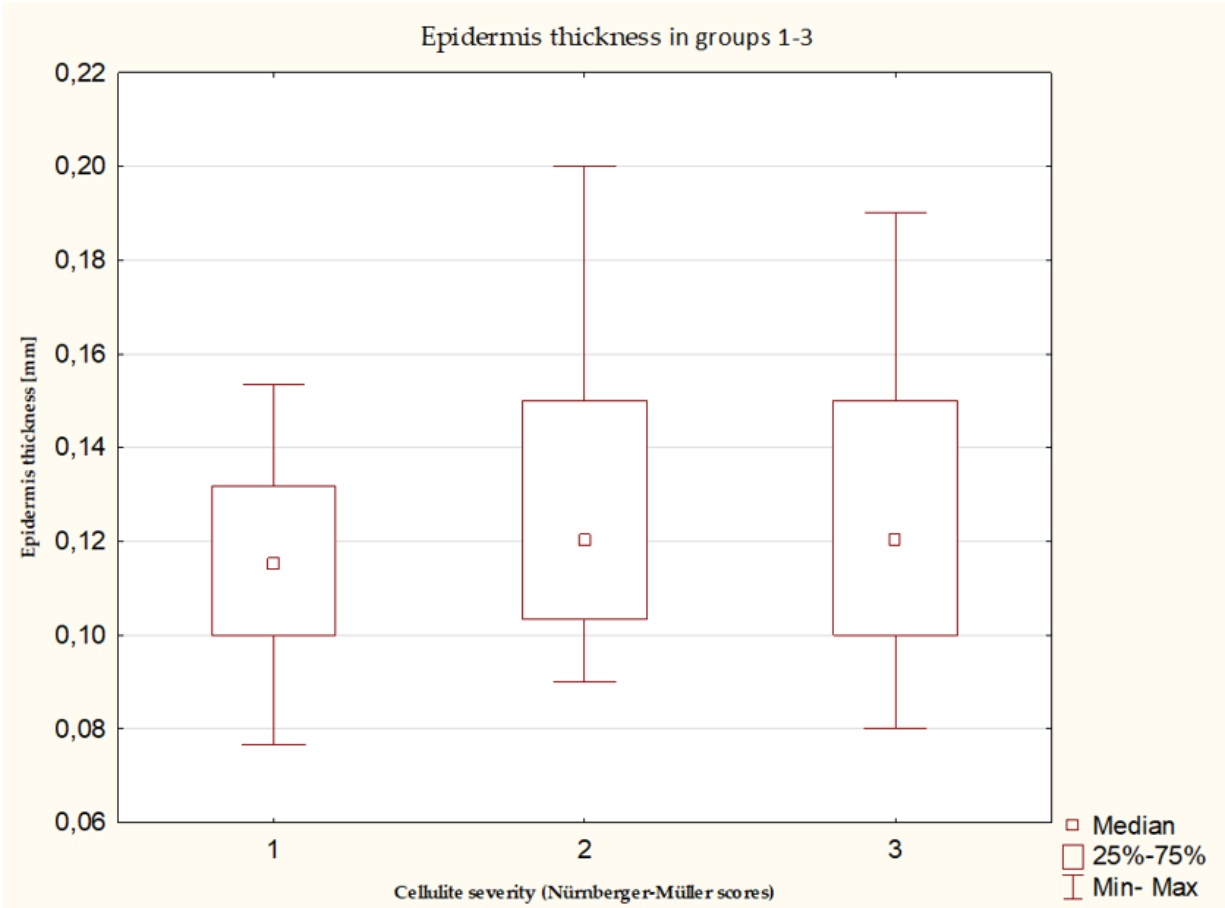

Supplement: Supplementary file 1 [file diagnostics-14-01878-s001.zip › Figure S1.jpg]

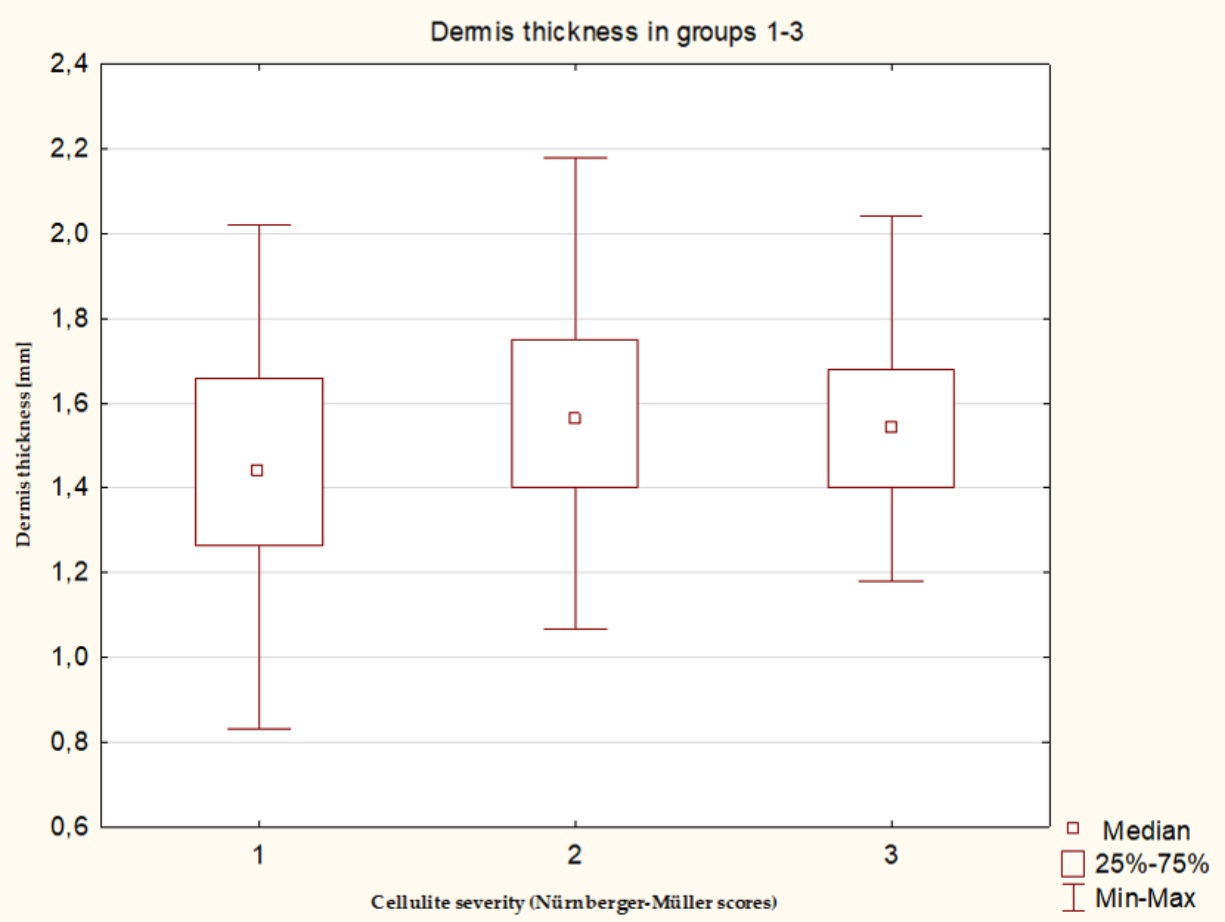

Supplement: Supplementary file 1 [file diagnostics-14-01878-s001.zip › Figure S2.jpg]

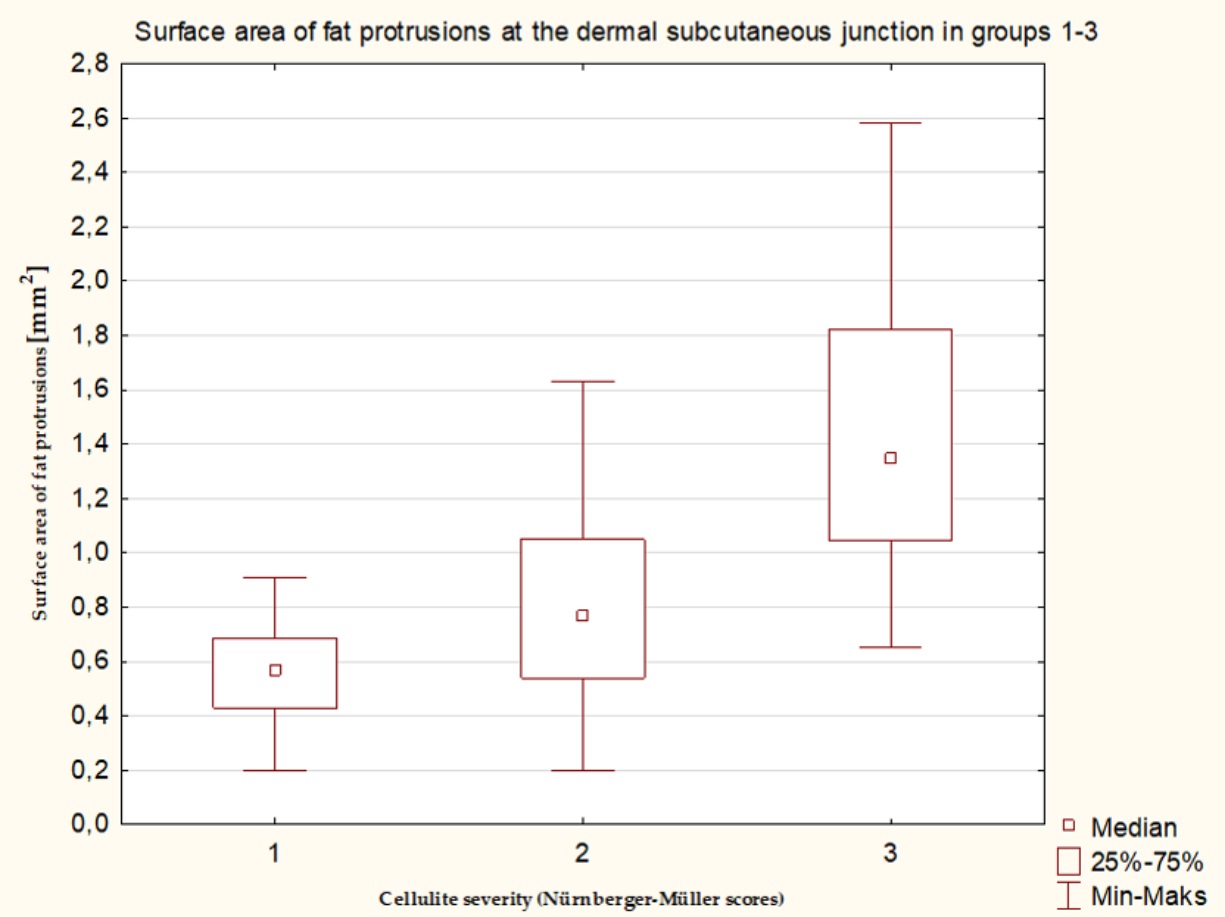

Supplement: Supplementary file 1 [file diagnostics-14-01878-s001.zip › Figure S3.jpg]

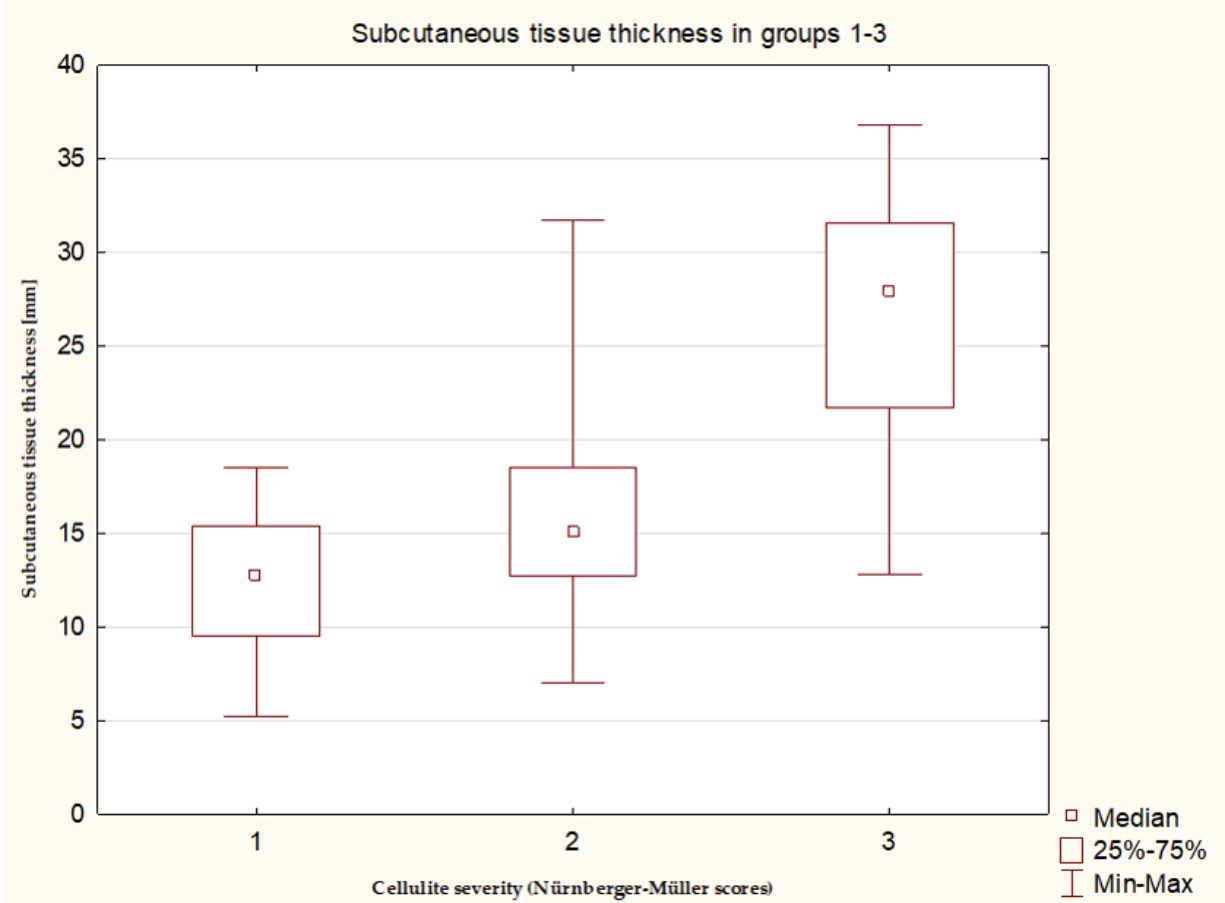

Supplement: Supplementary file 1 [file diagnostics-14-01878-s001.zip › Figure S4.jpg]

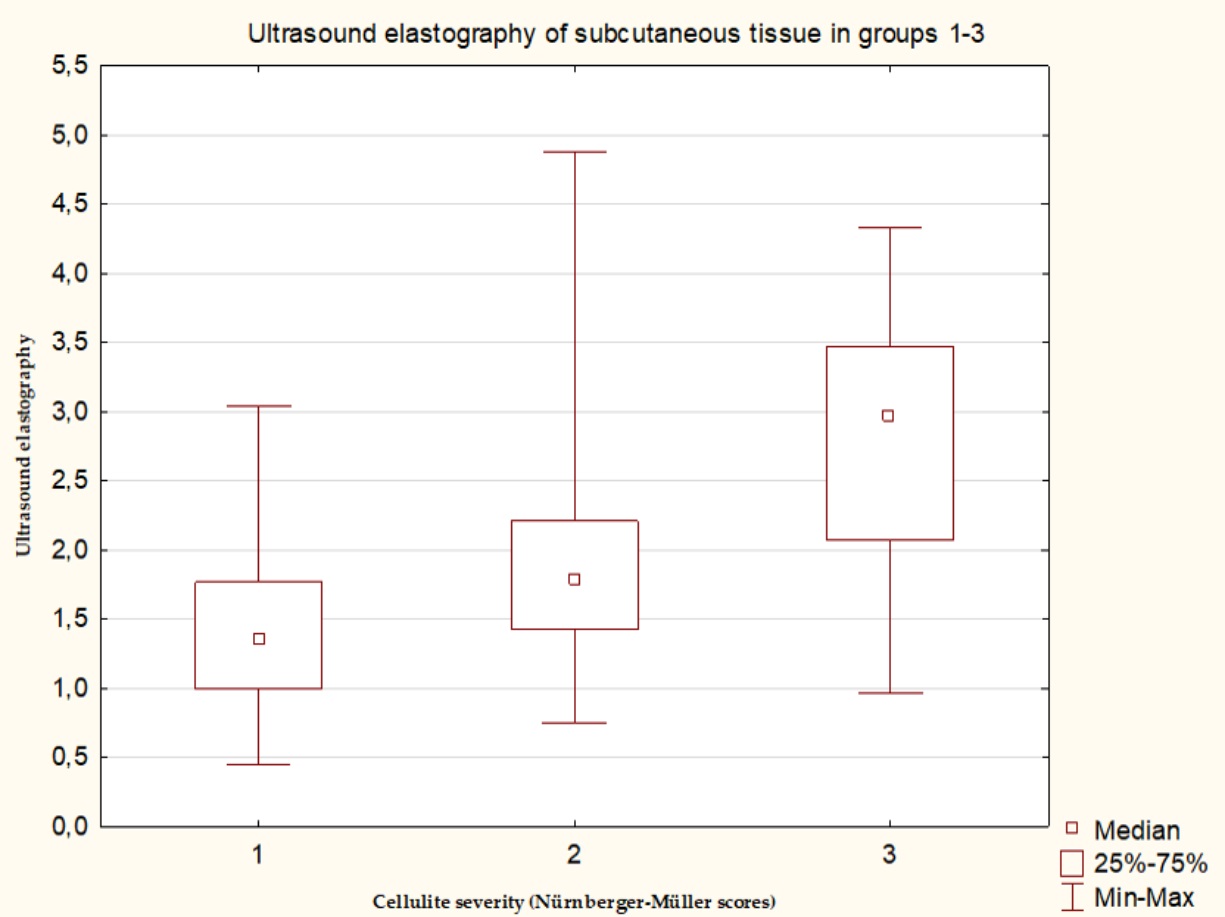

Supplement: Supplementary file 1 [file diagnostics-14-01878-s001.zip › Figure S5.jpg]
